# Hemodialysis Serum Stimulates the TXNIP-eNOS-STAT3 Inflammatory Pathway In Vitro

**DOI:** 10.3390/antiox12051109

**Published:** 2023-05-17

**Authors:** Keren Cohen-Hagai, Hadil Kashua, Sydney Benchetrit, Tali Zitman-Gal

**Affiliations:** 1 Department of Nephrology and Hypertension, Meir Medical Center, Kfar Saba 44281, Israel; 2 Sackler Faculty of Medicine, Tel Aviv University, Tel Aviv 69978, Israel; 3 Department of Pediatric, Meir Medical Center, Kfar Saba 44281, Israel

**Keywords:** hemodialysis, inflammatory response, HUVEC, TXNIP, eNOS, STAT3

## Abstract

Background: Endothelial dysfunction, vascular inflammation and accelerated atherosclerosis have been investigated extensively in patients with chronic kidney disease (CKD). These conditions, as well as protein–energy malnutrition and oxidative stress, impair kidney function and contribute to increased morbidity and mortality among patients with end-stage kidney disease undergoing hemodialysis (HD). TXNIP, a key regulator of oxidative stress, has been linked to inflammation and suppresses eNOS activity. STAT3 activation adds to endothelial cell dysfunction, macrophage polarization, immunity and inflammation. Therefore, it is critically involved in atherosclerosis. This study evaluated the effect of sera from HD patients on the TXNIP-eNOS-STAT3 pathway using an in vitro model of human umbilical vein endothelial cells (HUVECs). Methods: Thirty HD patients with end-stage kidney disease and ten healthy volunteers were recruited. Serum samples were taken at dialysis initiation. HUVECs were treated with HD or healthy serum (10% *v*/*v*) for 24 h. Then, cells were collected for mRNA and protein analysis. Results: TXNIP mRNA and protein expression were significantly increased in HUVECs treated with HD serum compared to healthy controls (fold changes: 2.41 ± 1.84 vs. 1.41 ± 0.5 and 2.04 ± 1.16 vs. 0.92 ± 0.29, respectively), as were IL-8 mRNA (fold changes: 2.22 ± 1.09 vs. 0.98 ± 0.64) and STAT3 protein expression (fold changes: 1.31 ± 0.75 vs. 0.57 ± 0.43). The expression of eNOS mRNA and protein (fold changes: 0.64 ± 0.11 vs. 0.95 ± 0.24; 0.56 ± 0.28 vs. 4.35 ± 1.77, respectively) and that of SOCS3 and SIRT1 proteins were decreased. Patients’ nutritional status, reflected by their malnutrition–inflammation scores, did not affect these inflammatory markers. Conclusions: This study showed that sera from HD patients stimulated a novel inflammatory pathway, regardless of their nutritional status.

## 1. Introduction

The worldwide prevalence of chronic kidney disease (CKD) is estimated to be 8–16%. It affects about 45% of people over the age of 70 years in the United States. CKD accounts for a substantial portion of the global health burden and is associated with increased morbidity and mortality [1]. CKD is expected to increase over time with the aging of the population and the increasing prevalence of diabetes and hypertension and is associated with adverse outcomes. The adverse outcomes of patients with CKD include cardiovascular events and increased mortality. Grams et al. [2] predicted a 1-year probability of 3.3% for developing cardiovascular disease in 60-year-old white women with stage 3B CKD and proteinuria, even in the absence of diabetes. The 1-year predictions for developing end-stage kidney disease (ESKD) and death in this model were 4.1% and 0.3%, respectively. These adverse outcomes increase as CKD progresses and are even higher among patients with ESKD treated with hemodialysis (HD). The pathophysiology is complicated and includes increased oxidative stress and sympathetic activity that coexist with disturbances in fluid balance, acid–base and electrolyte homeostasis, anemia and mineral bone disorder and the accumulation of uremic toxins. These contribute to endothelial dysfunction, vascular calcifications and accelerated atherosclerosis [3]. Endothelial dysfunction is pivotal in the pathogenesis of atherosclerosis and cardiovascular disease among patients with ESKD. Since normal vascular endothelium is critical in the prevention of atherosclerosis, it is not surprising that endothelial injury and dysfunction occur early in CKD and substantially contribute to cardiovascular disease in this population. Endothelial dysfunction in CKD is a result of multifactorial endothelial injuries due to uremic toxins and the consequences of the uremic milieu, such as advanced glycosylation products, low vitamin D and Klotho levels and increased levels of FGF23 [4]. Protein–energy malnutrition and inflammation also increase as CKD progresses and are associated with accelerated atherosclerosis and increased mortality. The prevalence of inflammation and protein malnutrition is highest among patients with ESKD undergoing HD. Both conditions are often found together in HD patients and accelerate atherosclerosis in this unique population. Therefore, they have been termed “malnutrition-inflammation-atherosclerosis syndrome” to highlight their association with atherosclerotic cardiovascular disease. Uremic toxins, diminished appetite, nausea and vomiting, dialysis-related nutrient loss and increased protein catabolism contribute to the malnutrition that aggravates existing inflammation, which accelerates atherosclerosis and vascular calcification. This increases the risk of cardiovascular disease and infections in this population. Several scores have been developed and validated to assess the nutritional status of these patients [5,6,7]. Malnutrition and inflammation are closely interrelated and together contribute to vascular calcification [8]. Soluble biomarkers of endothelial dysfunction and oxidative stress are increased among patients with CKD, and endothelium-dependent vasodilatation is affected, as well [9]. Endothelial dysfunction is characterized by impaired endothelium-dependent vasorelaxation and thereby reduces endothelial nitric oxide (eNOS)-derived NO bioactivity [10]. The key role of thioredoxin-interacting protein (TXNIP) is to regulate glucose and lipid metabolism and initiate inflammation and apoptosis [11]. Several tissues and cells express TXNIP, which endogenously suppresses thioredoxin (a reactive oxygen species scavenging protein). It is also an important sensor of oxidative inflammation and stress on a molecular level. [11,12]. It has been suggested that TXNIP can suppress eNOS activity and disrupt NO signaling, which indicates that it may have a detrimental effect on endothelial function [11]. He et al. [12] reported that in an in vitro model of tubular epithelial cells, TXNIP overexpression led to the upregulation of senescence markers, increased the profibrotic response and activated signal transducer and activator of transcription 3 (STAT3). TXNIP silencing inhibited these factors. The activation of STAT3 has a role in endothelial cell dysfunction, macrophage polarization, immunity and inflammation. As such, it might be a necessary modulator in the atherosclerotic process [13]. Using in vitro and in vivo models, our group demonstrated that the expression of endothelial and vascular inflammatory-related genes such as *TXNIP* and *STAT3* was increased and eNOS expression was decreased when subjected to diabetic-like conditions [14,15,16].

The goal of this study was to investigate the effect of sera from HD patients on the inflammatory markers TXNIP, eNOS and STAT3 using an in vitro model of human umbilical vein endothelial cells (HUVECs) and to assess the association of the nutritional–inflammatory state of HD patients with the expression pattern of these inflammatory markers.

## 2. Methods

### 2.1. Study Population

Inclusion criteria were adults (≥18 years) undergoing HD who were recruited to the study from 1 January 2022 to 1 December 2022. Only patients with previous chronic kidney disease that reached end-stage kidney disease and required renal replacement therapy by hemodialysis were included. Patients with acute kidney injury and patients who were treated with peritoneal dialysis were not included in this study. Healthy volunteers served as controls.

The nutritional–inflammatory state of each HD patient was assessed using the validated malnutrition–inflammation score (MIS) [17]. Both severely malnourished and stable ambulatory (“steady-state”) patients were included in the study to obtain a wide range of MIS values. The control group declared that they were healthy with no known comorbidities or chronic diseases. Data abstracted from the electronic medical record database included sex, age, baseline laboratory values and comorbidities. The results are reported according to the STROBE statement guidelines [18].

### 2.2. Ethics

This study was approved by the Institutional Ethics Committee of Meir Medical Center on 15 November 2021 (approval nos. 0192-21-MMC and 0074-11-MMC). Written informed consent was obtained from all patients. The Declaration of Helsinki and Good Clinical Practice guidelines were adhered to during the study.

### 2.3. Malnutrition–Inflammation Score

A semi-quantitative scale was used to evaluate 10 MIS components, including medical history, physical exam, body mass index (BMI) and laboratory parameters (serum albumin and total iron-binding capacity) based on Kalantar-Zadeh scoring criteria [17]. Each component was assigned a severity score ranging from 0 (normal) to 3 (very severe). The final scores ranged from 0 to 30, based on the total of the ten individual scales. Patients were grouped according to MIS scores of ≤12 (normal-to-mild malnutrition) or ≥13 (severe malnourishment).

### 2.4. Blood Samples

For the in vitro experiments, blood samples from HD patients and healthy volunteers were collected. The samples were taken from patients at dialysis initiation at the Dialysis Unit in Meir Medical Center. Serum was collected using low-speed centrifugation (3000× *g* for 10 min at 4 °C) and frozen immediately at −80 °C.

### 2.5. Endothelial Cell Culture

After obtaining Ethics Committee approval (0074-11-MMC) and written informed consent from parturients, endothelial cells were collected from HUVECs. Umbilical cords were obtained from healthy women without comorbidities who completed a normal uncomplicated pregnancy at 38–40 weeks of gestation. The cord veins were rinsed with phosphate-buffered saline (PBS) (Biological Industries, Beit Ha’emek, Israel), filled with collagenase H (0.3%; Merck, Darmstadt, Germany) in PBS and incubated for 10 min at 37 °C. The collagenase was removed, and the veins were washed with PBS. After centrifugation for 10 min at 1200 rpm, the HUVECs were collected and seeded into culturing flasks. The HUVECs were grown under standard cell culture conditions (humidified 5% CO_2_ atmosphere at 37 °C) in M-199 medium (Biological Industries, Beit Ha’emek, Israel) supplemented with 20% fetal calf serum (FCS), 100 U/mL penicillin, 100 µ/mL streptomycin and 0.25 µg/mL amphotericin, 5 U/mL heparin and 25 µg/mL endothelial mitogen (Millipore, Temecula, CA, USA).

### 2.6. HUVEC Treatment

Confluent cultures of HUVECs from eight different donors at passages 2–4 were used for experiments. Each cell culture was used for an individual experiment. Subconfluent HUVECs were serum-deprived for 24 h to obtain cell synchronization and then pooled with human sera (10% *v*/*v*) obtained from HD patients (*n* = 30) or from healthy volunteers (*n* = 10). We randomly divided the sera collected from HD patients into five groups, with 4 to 6 samples in each pool used for the HUVEC treatment. When analyzing according to MIS groups, sera were divided according to MIS scores of ≤12 (normal-to-mild malnutrition) or ≥13 (severe malnourishment). FCS was used for control HUVECs. After 24 h of incubation, the cells were trypsinized for mRNA and protein analysis to identify the selected inflammatory markers.

### 2.7. STRING Database

**Figure 1:**
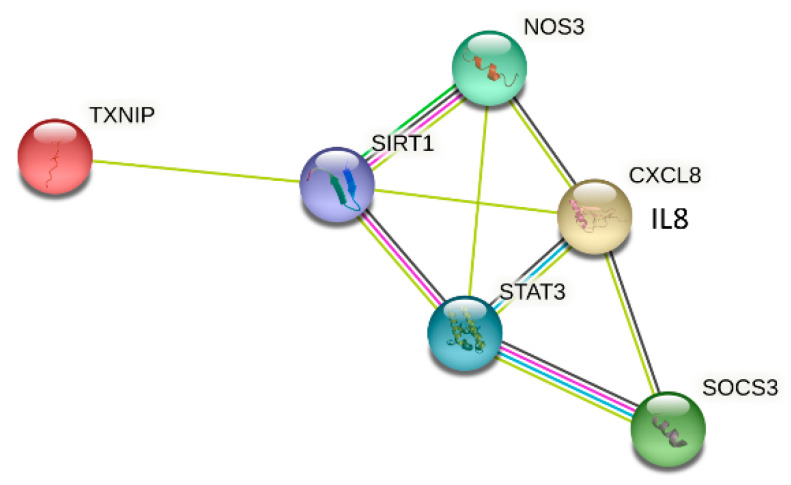
Protein biological interactions. The String database was used to analyze predicted protein–protein interactions of TXNIP, SIRT1, SOCS3, STAT3, eNOS (NOS3) and IL-8 (CXCL8). The colored lines represent interactions between proteins (blue—interaction from curated database; pink—experimentally determined; yellow—text mining; and gray—co-expression) (https://string-db.org). Associations are meant to be specific and meaningful; i.e., proteins jointly contribute to a shared function.

STRING is a proteomic database focusing on the networks of known and predicted protein–protein interactions. STRING allows searches for one or several proteins simultaneously, with the ability to limit the search to the desired species. It includes direct (physical) and indirect (functional) associations derived from various sources, such as genomic contexts, high-throughput experiments, (conserved) co-expression and the literature. We used the STRING database (https://string-db.org/) to present the selected inflammatory proteins TXNIP, eNOS, STAT3, IL-8, SIRT1 and SOCS3 for this research (Figure 1).

### 2.8. RNA Extraction and Reverse Transcription (RT)

The RNeasy mini kit (QIAGEN, Hilden, Germany) was used to extract RNA from HUVECs according to the manufacturer’s instructions. RNA was then reverse-transcribed into single-stranded DNA using the High-Capacity cDNA Reverse Transcription Kit (Applied Biosystems Inc., Foster City, CA, USA) according to the manufacturer’s instructions.

### 2.9. Real-Time Polymerase Chain Reaction (PCR)

Real-time polymerase chain reaction (PCR) (7500 Fast Real Time PCR System, Applied Biosystems, Inc. Thermo Fisher Scientific, Waltham, MA, USA) was performed using SYBER Green I reaction mix (Applied Biosystems Inc., Thermo Fisher Scientific, Waltham, MA, USA). The thermal profile for SYBR Green PCR was a holding stage at 95 °C for 20 s, followed by 40 cycles at 95 °C for 3 s and at 60 °C for 30 s. A melting curve analysis was performed to ensure the specificity of the real-time PCR reaction. Data were analyzed using the 2^−ΔΔCt^ method. Individual primers for TXNIP, IL-8, eNOS and glucuronidase beta (GUSB) were used, as previously described [14,15,16]: TXNIP primers: forward primer 5′-AGATCAGGTCTAAGCAGCAGAACA-3′; reverse primer 5′-CCATATAGCAGGGAGGAGCTTC-3′. eNOS primers: forward primer 5′-TGCAGTTGCTGCCAGGTC-3′; reverse primer 5′-GGACTTGCTGCTTTGCAGGT-3′. IL-8 primers: forward primer 5_-CTCTTGGCAGCCTTCCTGATTT-3; reverse primer 5_-TGGGGTGGAAAGGTTTGGAGTA-3. GUSB primers: forward primer 5′-CAATACCTGACTGACACCTCCAGTA-3′; reverse primer 5′-TGGTGGGTGTCGTGTACAGAAGT-3′.

### 2.10. HUVEC Protein Extraction

HUVECs were trypsinized, and the pellet was washed with PBS. Next, the cells were lysed in 50 μL of lysis buffer (1X RIPA Buffer, Abcam, Cambridge, MA, USA). The tubes were kept on ice for 10 min and centrifuged at 13,000× *g* for 10 min at 4 °C. The supernatant was collected into new microtubes. The BCA^TM^ protein assay kit (Thermo Scientific, Rockford, IL, USA) was used to determine protein concentrations.

### 2.11. SDS-PAGE and Immunoblotting

Samples were separated by SDS-PAGE using 10% polyacrylamide gels and transferred to nitrocellulose filters. Blots were blocked for 1 h in TBST (10 mM Tris-HCl (pH 8.0), 150 mM NaCl and 0.05% Tween 20) containing 5% skim milk, followed by overnight incubation at 4 °C with the indicated primary antibodies. The following monoclonal antibodies were used: anti-TXNIP (1:500 Abcam), anti-eNOS (1:500, BD Transduction Laboratories, NJ, USA) and anti-α-tubulin (1:8000, Sigma-Aldrich, Detroit, MI, USA), as well as SIRT1 and SOCS3 (both 1:500 Abcam) and anti-phospho-STAT-3 (1:1000, Cell Signaling Technology, Danvers, MA, USA). Blots were washed three times with TBST and incubated for 1 h at room temperature with HRP-conjugated secondary antibodies. Before reprobing with a different antibody, the nitrocellulose membranes were stripped and blocked with skim milk. The Enhanced Chemiluminescent Reporter System (Millipore) was used to visualize the bound antibodies. Protein expression was quantified using LAS-3500 (Fujifilm Corp., Tokyo, Japan). The expression of the TXNIP protein was detected as a single band at 50 kDa, and the expression of the eNOS protein was detected as a single band at 140 kDa. P-STAT3 was detected as a single band at 86 kDa, SOCS3 was detected as a single band at 25 kDa, SIRT1 was detected as a single band at 120 kDa, and α-tubulin, as the normalized control, was detected as a single band at 50 kDa. The quantification of protein expression was normalized against the quantification of α-tubulin.

### 2.12. Human IL-8 Assay

Supernatants obtained from the different culture conditions of HUVECs and human sera described in Section 2.6 were examined for the release of IL-8 using a commercial ELISA kit (DuoSet; Peprothech, Cranbury, NJ, USA).

### 2.13. Statistical Analysis

Data are expressed as absolute numbers (%) or as medians with interquartile ranges. Normality was tested using the Shapiro–Wilk test. Continuous variables are shown as medians with interquartile ranges. MIS was calculated as previously described [19]. One-way ANOVA was used to compare independent samples. Pearson and Spearman correlation tests were applied, each as appropriate. The Pearson correlation was used to assess a linear relationship between two continuous variables, and Spearman correlation tests were applied to all the correlation tests for the other variables. *p*-values < 0.05 were considered statistically significant. All statistical analyses were performed using SPSS-28 (IBM, Armonk, NY, USA).

## 3. Results

### 3.1. Study Sample

The study cohort included 30 HD patients and 10 healthy volunteers, who served as controls. The control group included healthy volunteers, aged 18–50 years. The mean age was 33.8 ± 10.3 years, and 50% were male.

The characteristics of HD patients are shown in Table 1. Most were men (27, 90%). Comorbidities included diabetes in 63% and hypertension in 93%. The median MIS score was 5, and 25 (83%) patients had a relatively low MIS of <12. Positive Pearson correlations (r) were found between nutritional factors, such as serum albumin with creatinine (r = 0.6, *p* < 0.01), hemoglobin (r = 0.4, *p* = 0.03), folic acid (r = 0.5, *p* < 0.01) and phosphorus (r = 0.4, *p* = 0.05). Albumin and the inflammatory marker CRP were inversely correlated (−0.5, *p* = 0.01), as were albumin and MIS (−0.7, *p* < 0.01).

### 3.2. The Effect of HD Serum on TXNIP, eNOS and IL-8 mRNA Expression

**Figure 2:**
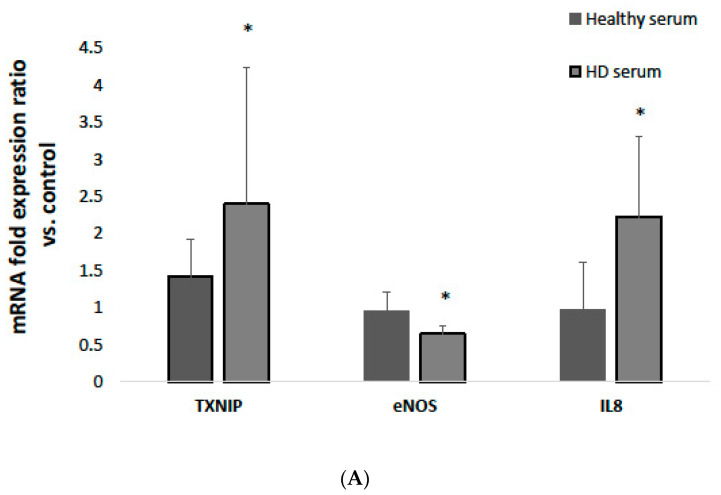

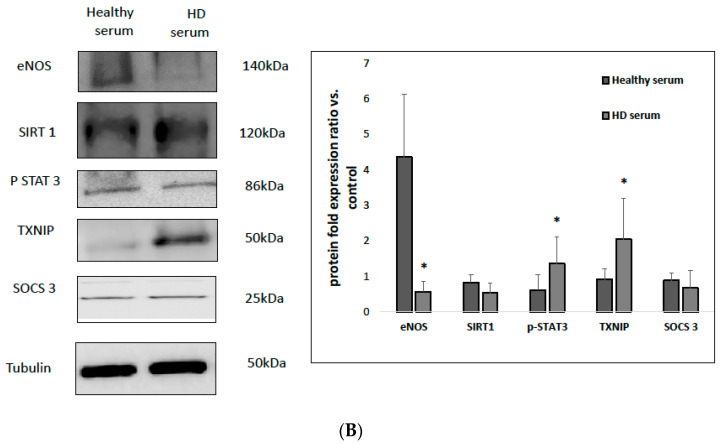
mRNA and protein expression of inflammatory markers isolated from HUVECs treated with HD and healthy sera. (**A**) TXNIP, eNOS and IL-8 mRNA fold expression ratio vs. control (untreated HUVECs). (**B**) TXNIP, eNOS, STAT3, SOCS3 and SIRT1 protein expression presented in Western blot and densitometry analyses (fold expression was calculated vs. control untreated HUVECs). Data are the mean ± SD of 8 independent experiments of cultured HUVEC. * *p* < 0.05 compared with healthy sera.

We investigated the effect of sera obtained from HD patients on the mRNA expression of selected inflammatory markers in an in vitro model of HUVECs. We found that TXNIP mRNA expression was significantly increased in HUVECs treated with HD serum compared to healthy controls (2.41 ± 1.84 vs. 1.41 ± 0.5, respectively, *p* < 0.05; Figure 2). eNOS mRNA expression was significantly decreased in HUVECs treated with HD serum compared to healthy controls (0.64 ± 0.11 vs. 0.95 ± 0.24, respectively, *p* < 0.05; Figure 2A). IL-8 mRNA expression was increased in HUVECs treated with HD serum compared to healthy controls (2.22 ± 1.09 vs. 0.98 ± 0.64; *p* < 0.05 respectively; Figure 2A). The analysis of TXNIP, eNOS and IL-8 mRNA expression according to MIS ≤ 12 (normal-to-mild malnutrition) vs. ≥13 (severe malnourishment) revealed no significant differences.

### 3.3. The Effect of HD Serum on TXNIP, eNOS, STAT3, SOCS3, SIRT1 and IL-8 Protein Expression

We investigated the effect of sera obtained from HD patients on the protein expression of the selected inflammatory markers in an in vitro model of HUVECs. We found that the protein expression of TXNIP and STAT3 was increased in HUVECs treated with sera from HD patients compared to sera from healthy controls (TXNIP: 2.04 ± 1.16 vs. 0.92 ± 0.29, respectively, *p* < 0.01; STAT3: 1.31 ± 0.75 vs. 0.57 ± 0.43, respectively, *p* < 0.01; Figure 2B). In contrast, the protein expression of eNOS, SOCS3 and SIRT1 was decreased in HUVECs treated with sera from HD patients compared to sera from healthy controls (eNOS: 0.56 ± 0.28 vs. 4.35 ± 1.77, respectively, *p* < 0.01; SOCS3: 0.67 ± 0.08 vs. 0.89 ± 0.41, respectively, *p* = 0.22; and SIRT1: 0.53 ± 0.48 vs. 0.82 ± 0.2, *p* = 0.07; Figure 2B). No significant increase in IL-8 protein secretion was found in HD-serum-treated HUVECs compared to healthy-serum-treated HUVECs. Moreover, there were no significant differences in TXNIP, eNOS, STAT3, SOCS3, SIRT1 or IL-8 protein expression when analyzed according to MIS ≤ 12 (normal-to-mild malnutrition) vs. ≥13 (severe malnourishment).

## 4. Discussion

This study evaluated the effect of sera from HD patients on endothelial inflammation in an in vitro model of HUVECs. The novel findings indicate that sera from HD patients stimulated the TXNIP-eNOS-STAT3 endothelial inflammatory response in the in vitro model. The malnutrition–inflammation status of hemodialysis patients did not significantly affect the stimulation of the TXNIP-eNOS-STAT3 endothelial inflammatory response. This could be due to the small cohort or could be explained by the influence of other contributing factors that were not assessed in this study.

Patients with uremia are a heterogeneous population, and the wide range of dialysis vintages in such a small cohort may mask the effect of uremic toxins and nutritional status on endothelial cell function. Therefore, we assessed the nutritional and inflammatory states of each patient using a validated score that clearly defines their status and has prognostic significance among this unique population.

Endothelial dysfunction (ED), the basis of atherosclerosis, progressively increases with ESKD. Nitric oxide (NO) is a gaseous molecule with vasorelaxant, anti-inflammatory and antithrombotic properties. One sign of ED is the decreased bioavailability of NO [20]. NO has a major role in preventing ED and arterial remodeling. It inhibits platelet aggregation and the adhesion of monocytes to endothelial cells, negates the oxidation of low-density lipoprotein cholesterol and suppresses smooth muscle cell hyperplasia and hypertrophy [20]. In the current study, after 24 h of exposure to HD serum, eNOS was decreased in endothelial cells compared to cells treated with the sera from healthy volunteers. Additionally, TXNIP, STAT3 and IL-8 expression were increased, which implies increased endothelial cell inflammation.

Overexpression of TXNIP along with thioredoxin impedes its antioxidant functions, which increases oxidative stress [21]. TXNIP overexpression in endothelial cells from human aortas, induced by high glucose, directly caused apoptosis and impaired NO function [22]. In this study, we showed that TXNIP is also upregulated after stimulation with HD serum and probably contributes to the development of ED.

Sun et al. [23] investigated the association between eNOS and TXNIP and showed that trimethylamine N-oxide caused inflammation and ED by activating the ROS-TXNIP-NLRP3 inflammasome, while eNOS and NO production was inhibited. Using an endothelial-specific TXNIP overexpression (EKI) mouse model, Lam et al. [24] demonstrated that inducing endothelial TXNIP resulted in impaired vasorelaxation in EKI mice, and this was related to a decrease in Akt and eNOS activation, along with increased NLRP3 activation.

He et al. [12] used TXNIP-overexpressing and TXNIP-silenced cells to confirm the role of the STAT3 signaling pathway in the TXNIP-mediated cellular profibrotic response. They found an increase in STAT3 expression in the TXNIP-overexpressing human proximal tubular epithelial cells (HK-2) and primary renal proximal tubule epithelial cells (PTEC) compared with the control cells, whereas lower levels of STAT3 phosphorylation were detected in the TXNIP-silenced cells. They used STAT3-specific inhibitors and found decreased expression of activated STAT3 and decreased TGF-β1/Smad3 signaling, which resulted in the alleviation of TXNIP-mediated fibrosis. They concluded that TXNIP directly interacts with STAT3 in tubular cells infected with TXNIP.

An increasing number of studies have demonstrated that the JAK2/STAT3 signaling pathway is strongly activated when modulating atherosclerosis, including endothelial cell dysfunction, macrophage polarization, inflammation and immunity [13]. STAT3 functions as an essential signal transduction effector protein for cytokine- and hormone-induced pathways that control the development, proliferation or differentiation, and homeostasis of numerous cell types [13]. In cytokine-dependent transcription, STAT3 mediates the primary response through the formation of p-STAT3 dimers. It plays a secondary role in the complete response by increasing the amounts of unphosphorylated STAT3 [13]. SOCS3 is associated with the negative regulation of STAT3 activation, which limits STAT3 phosphorylation. Oxidative stimulation slows the activation rate of SOCS3 and causes the mislocalization of the STAT3 phosphatase TC-PTP to the cytoplasm, both of which diminish their ability to inhibit the phosphorylation of STAT3 [25]. The phosphorylation of STAT3 can activate Ras homolog gene family member A (RhoA), which inhibits eNOS phosphorylation [13]. eNOS suppression promotes ICAM-1 and VCAM-1 expression, which can decrease their stability and result in more cells adhering to plaques, which leads to ED [13].

The current study demonstrated that p-STAT3 expression is increased in HUVECs exposed to HD serum, while SOCS3 and eNOS expression were diminished. This expression pattern reflects the impact of HD on the progression of ED.

The study results also showed decreased SIRT1 expression. Sirtuins include seven highly conserved mammalian proteins that are involved in the homeostasis of cellular energy [26]. Among them, SIRT1 is a well-defined enzyme that responds to caloric restriction, starvation and stress. It serves as a key regulator in vascular endothelial homeostasis by controlling angiogenesis and decreasing atherosclerosis and endothelial dysfunction [26,27,28]. In a rat-based study, Hirabayashi et al. [28] found that malnutrition resulted in the elevated expression of phosphorylated AMPK in the soleus and plantaris muscles. In contrast, in the malnourished group, despite increased AMPK expression in both muscles, SIRT1 protein expression decreased. Additional studies showed that oxidative stress activates AMPK [29,30,31] but inhibits SIRT1. It has been hypothesized that there is crosstalk between SIRT1 and STAT3 during oxidative stress [32,33,34] and that SIRT1 exerts negative effects on the regulation of STAT3 expression. Therefore, it seems that SIRT1 only regulates the transcription response and promotes the state of ED.

CKD is common and is expected to increase with the aging of the population and the increasing prevalence of comorbidities that are associated with CKD, such as hypertension, diabetes and obesity. This increasing prevalence highlights the need for further research investigating the unique pathogenesis of inflammatory pathways in this population. The clinical implications of research into the complex, prevalent disorder of CKD, with its significant morbidity and mortality, are extensive. This study focused on ESKD patients because they represent extreme CKD. The comparison to healthy volunteers, who served as controls, highlights the differences. The increased inflammatory process, along with accelerated atherosclerosis, followed by increased morbidity and mortality, should encourage further research in this area. Ciceri and his group [35] found that several uremic retention solutes were significantly correlated with inflammation, malnutrition and atherosclerosis/calcification. This supports the hypothesis that the uremic milieu has a central role in malnutrition–inflammation–atherosclerosis syndrome and ultimately in the pathogenesis of specific CKD risk factors. The uremic milieu is characterized by the accumulation of biologically active molecules in the bloodstream. Normally, these uremic toxins would be cleared by healthy kidneys through the urine [36,37]. Since an inflammatory state is present in CKD and progresses as kidney function deteriorates [38,39], we believe that the TXNIP-eNOS-STAT3 pathway might also be interrupted in earlier stages of CKD. The effect of uremic toxins and other variables (such as bone mineral parameters) and comorbidities, such as diabetes, that contribute to oxidative stress should also be considered. Inflammation, oxidative stress and ultimately fibrosis offer opportunities to positively manipulate the cascade and perhaps even ameliorate the deleterious consequences of renal diseases and cardiovascular morbidity found in this population. This should encourage further research in the field.

Additional information on the clinical implications of studies combining in vivo and in vitro models, along with validated, clinically objective assessments of patients, such as the MIS score and the STRING database used in this study, is strongly needed.

This study had a few limitations. In addition to the small sample size, due to the possible selection bias of a single HD center, the sample might not represent the general population of uremic patients. Some patients had comorbidities, such as diabetes mellitus, that are known to affect ED. The population of HD patients was heterogeneous, and there may have been other pathologies and confounding variables that cannot be tested in a small study. In this study, healthy volunteers served as controls, and they were not matched by age and sex to the study group. However, the use of healthy controls without known comorbidities highlights the unique effect of uremic serum on the TXNIP-eNOS-STAT3 pathway. This has not been described previously.

## 5. Conclusions

This study showed for the first time that, regardless of their nutritional status, sera from HD patients stimulated an inflammatory response in vitro, represented by the TXNIP-eNOS-STAT3 markers. Further studies that analyze the association between HD uremic serum and biomarkers of malnutrition–inflammation–atherosclerosis syndrome are needed.

## Figures and Tables

**Table 1:** Baseline characteristics of HD patients (*N* = 30).

Characteristic	Median [IQR]
Age (years)	67.8 [57.1–78.5]
Male sex	27 (90)
Body mass index	27.6 [23.6–31.3]
Malnutrition–inflammation score (MIS)	5 [2.75–7]
Dialysis vintage (months)	22 [2–43.5]
Weekly dialysis hours	12 [12–12]
Ischemic heart disease	12 (40)
Heart failure	7 (23)
Hypertension	28 (93)
Diabetes mellitus	19 (63)
Chronic lung disease	7 (23)
Previous stroke	6 (20)
Active/past malignancy	5 (17)
Atrial fibrillation	6 (20)
Peripheral vascular disease	5 (17)
Left ventricular hypertrophy	12 (40)
Active smoking	10 (33)
Baseline laboratory values	
Serum creatinine (mg/dL)	6.42 [5.3–8.6]
Serum urea (mg/dL)	115 [98–146]
Albumin (gr/dL)	3.8 [3.5–4.1]
Total cholesterol (mg/dL)	153 [128–183]
Calcium (mg/dL)	8.6 [8.3–8.9]
Phosphorus (mg/dL)	5.2 [4.4–6]
Parathyroid hormone (pg/mL)	285 [170–580]
Hemoglobin A1C (%)	6.1 [5.5–6.75]
Glucose (mg/dL)	111 [100–156]
C-reactive protein (mg/L)	1.1 [0.6–2.5]
White blood cells (K/µL)	6.6 [5.7–8.5]
Platelets (K/µL)	188 [151–225]
Hemoglobin (g/dL)	11.1 [10–11.5]
Transferrin saturation index	0.25 [0.19–0.29]
Iron (µg/dL)	64 [49–72]
Transferrin (mg/dL)	166 [158–199]
Ferritin (µg/L)	684 [319–880]
B12 (ng/L)	508 [436–895]
Folic acid (µg/L)	20 [4–20]
25(OH) VitD (nmol/L)	52.3 [32.6–66.3]
kt/v	1.32 [1.18–1.53]
Normalized protein catabolic rate (g/kg/day)	0.89 [0.76–1.11]
Urea reduction ratio	68.3 [64.5–74.2]

Data are presented as absolute numbers (%) or as median [IQR]; laboratory tests were performed at dialysis initiation. Malnutrition–inflammation score: ≤12 (normal-to-mild malnutrition); ≥13 (severely malnourished). 25(OH) VitD, 25-hydroxyvitamin D; kt/v, k-dialyzer clearance of urea; t, dialysis time; v, volume of distribution of urea, approximately equal to patient’s total body fluid volume.

## Data Availability

Data is contained within the article.

## Abbreviations

CKD	Chronic kidney disease
eNOS	Endothelial nitric oxide synthase
ESKD	End-stage kidney disease
HD	Hemodialysis
HUVEC	Human umbilical vein endothelial cells
IL-8	Interleukin 8
NO	Nitric oxide
SIRT1	Sirtuin 1
SOCS3	Suppressor of cytokine signaling 3
STAT3	Signal transducer and activator of transcription *3*
TXNIP	Thioredoxin-interacting protein

## References

[1] Sundström J., Bodegard J., Bollmann A., Vervloet M.G., Mark P.B., Karasik A., Taveira-Gomes T., Botana M., Birkeland K.I., Thuresson M. (2022). Prevalence, outcomes, and cost of chronic kidney disease in a contemporary population of 2·4 million patients from 11 countries: The CaReMe CKD study. Lancet Reg. Health Eur..

[2] Grams M.E., Yang W., Rebholz C.M., Wang X., Porter A.C., Inker L.A., Horwitz E., Sondheimer J.H., Hamm L.L., He J. (2017). Risks of Adverse Events in Advanced CKD: The Chronic Renal Insufficiency Cohort (CRIC) Study. Am. J. Kidney Dis..

[3] Podkowińska A., Formanowicz D. (2020). Chronic Kidney Disease as Oxidative Stress- and Inflammatory-Mediated Cardiovascular Disease. Antioxidants.

[4] Roumeliotis S., Mallamaci F., Zoccali C. (2020). Endothelial Dysfunction in Chronic Kidney Disease, from Biology to Clinical Outcomes: A 2020 Update. J. Clin. Med..

[5] Kalantar-Zadeh K., Ikizler T., Block G., Avram M.M., Kopple J.D. (2003). Malnutrition-inflammation complex syndrome in dialysis patients: Causes and consequences. Am. J. Kidney Dis..

[6] Rambod M., Bross R., Zitterkoph J., Benner D., Pithia J., Colman S., Kovesdy C.P., Kopple J.D., Kalantar-Zadeh K. (2009). Association of Malnutrition-Inflammation Score with quality of life and mortality in hemodialysis patients: A 5-year prospective cohort study. Am. J. Kidney Dis..

[7] Maraj M., Kuśnierz-Cabala B., Dumnicka P., Gala-Błądzińska A., Gawlik K., Pawlica-Gosiewska D., Ząbek-Adamska A., Mazur-Laskowska M., Ceranowicz P., Kuźniewski M. (2018). Malnutrition, Inflammation, Atherosclerosis Syndrome (MIA) and Diet Recommendations among End-Stage Renal Disease Patients Treated with Maintenance Hemodialysis. Nutrients.

[8] Gross M.-L., Meyer H.-P., Ziebart H., Rieger P., Wenzel U., Amann K., Berger I., Adamczak M., Schirmacher P., Ritz E. (2007). Calcification of coronary intima and media: Immunohistochemistry, backscatter imaging, and x-ray analysis in renal and nonrenal patients. Clin. J. Am. Soc. Nephrol..

[9] Jourde-Chiche N., Dou L., Cerini C., Dignat-George F., Brunet P. (2011). Vascular incompetence in dialysis patients--protein-bound uremic toxins and endothelial dysfunction. Semin. Dial..

[10] Kawashima S., Yokoyama M. (2004). Dysfunction of endothelial nitric oxide synthase and atherosclerosis. Arter. Thromb. Vasc. Biol..

[11] Wang R., Guob Y., Li L., Luo M., Peng L., Lv D., Cheng Z., Xue Q., Wang L., Huang J. (2020). Role of thioredoxin-interacting protein in mediating endothelial dysfunction in hypertension. Genes Dis..

[12] He Q., Li Y., Zhang W., Chen J., Deng W., Liu Q., Liu Y., Liu D. (2021). Role and mechanism of TXNIP in ageing-related renal fibrosis. Mech. Ageing Dev..

[13] Chen Q., Lv J., Yang W., Xu B., Wang Z., Yu Z., Wu J., Yang Y., Han Y. (2019). Targeted inhibition of STAT3 as a potential treatment strategy for atherosclerosis. Theranostics.

[14] Zitman-Gal T., Green J., Pasmanik-Chor M., Oron-Karni V., Bernheim J. (2010). Endothelial pro-atherosclerotic response to extracellular diabetic-like environment: Possible role of thioredoxin-interacting protein. Nephrol. Dial. Transplant..

[15] Einbinder Y., Ohana M., Benchetrit S., Zehavi T., Nacasch N., Bernheim J., Zitman-Gal T. (2016). Glucagon-like peptide-1 and vitamin D: Anti-inflammatory response in diabetic kidney disease in db/db mice and in cultured endothelial cells. Diabetes Metab. Res. Rev..

[16] Zitman-Gal T., Einbinder Y., Ohana M., Katzav A., Kartawy A., Benchetrit S. (2019). Effect of liraglutide on the Janus kinase/signal transducer and transcription activator (JAK/STAT) pathway in diabetic kidney disease in db/db mice and in cultured endothelial cells. J. Diabetes.

[17] Kalantar-Zadeh K., Kopple J.D., Humphreys M.H., Block G. (2004). Comparing outcome predictability of markers of malnutrition-inflammation complex syndrome in haemodialysis patients. Nephrol. Dial. Transplant..

[18] Vandenbroucke J.P., von Elm E., Altman D.G., Gøtzsche P.C., Mulrow C.D., Pocock S.J., Poole C., Schlesselman J.J., Egger M. (2007). STROBE Initiative. Strengthening the Reporting of Observational Studies in Epidemiology (STROBE): Explanation and elaboration. PLoS Med..

[19] Cohen-Hagai K., Nacasch N., Sternschuss A., Ohana M., Wolach B., Benchetrit S., Gavrieli R., Zitman-Gal T. (2020). Malnutrition and inflammation in hemodialysis patients: Comparative Evaluation of Neutrophil Reactive Oxygen Formation. Nutrition.

[20] Vanhoutte P.M., Zhao Y., Xu A., Leung S.W.S. (2016). Thirty Years of Saying NO: Sources, Fate, Actions, and Misfortunes of the Endothelium-Derived Vasodilator Mediator. Circ. Res..

[21] Qayyum N., Haseeb M., Kim M.S., Choi S. (2021). Role of Thioredoxin-Interacting Protein in Diseases and Its Therapeutic Outlook. Int. J. Mol. Sci..

[22] Tsubaki H., Tooyama I., Walker D.G. (2020). Thioredoxin-Interacting Protein (TXNIP) with Focus on Brain and Neurodegenerative Diseases. Int. J. Mol. Sci..

[23] Sun X., Jiao X., Ma Y., Liu Y., Zhang L., He Y., Chen Y. (2016). Trimethylamine N-oxide induces inflammation and endothelial dysfunction in human umbilical vein endothelial cells via activating ROS-TXNIP-NLRP3 inflammasome. Biochem. Biophys. Res. Commun..

[24] Lam Y.T., Tan R.P., Michael P., Yang N., Dunn L.L., Cooke J.P., Celermajer D.S., Wise S.G., Ng M.K.C. (2022). Endothelial thioredoxin interacting protein (TXNIP) modulates endothelium-dependent vasorelaxation in hyperglycemia. Microvasc. Res..

[25] Ng I.H., Yeap Y.Y., Ong L.S., Jans D.A., Bogoyevitch M.A. (2014). Oxidative stress impairs multiple regulatory events to drive persistent cytokine-stimulated STAT3 phosphorylation. Biochim. Biophys. Acta.

[26] Fang Y., Tang S., Li X. (2019). Sirtuins in Metabolic and Epigenetic Regulation of Stem Cells. Trends Endocrinol. Metab..

[27] Nogueiras R., Habegger K.M., Chaudhary N., Finan B., Banks A.S., Dietrich M.O., Horvath T.L., Sinclair D.A., Pfluger P.T., Tschöop M.H. (2012). Sirtuin 1 and sirtuin 3: Physiological modulators of metabolism. Physiol. Rev..

[28] Hirabayashi T., Nakanishi R., Tanaka M., Nisa B.U., Maeshige N., Kondo H., Fujino H. (2021). Reduced metabolic capacity in fast and slow skeletal muscle via oxidative stress and the energy-sensing of AMPK/SIRT1 in malnutrition. Physiol. Rep..

[29] Auciello F.R., Ross F.A., Ikematsu N., Hardie D.G. (2014). Oxidative stress activates AMPK in cultured cells primarily by increasing cellular AMP and/or ADP. FEBS Lett..

[30] Chen Z., Shentu T.-P., Wen L., Johnson D.A., Shyy J.Y.-J. (2013). Regulation of SIRT1 by oxidative stress-responsive miRNAs and a systematic approach to identify its role in the endothelium. Antioxid. Redox Signal..

[31] Liang D., Zhuo Y., Guo Z., He L., Wang X., He Y., Li L., Dai H. (2020). SIRT1/PGC-1 pathway activation triggers autophagy/mitophagy and attenuates oxidative damage in intestinal epithelial cells. Biochimie.

[32] Nie Y., Erion D.M., Yuan Z., Dietrich M., Shulman G.I., Horvath T.L., Gao Q. (2009). STAT3 inhibition of gluconeogenesis is downregulated by SirT1. Nat. Cell Biol..

[33] Bernier M., Paul R.K., Martin-Montalvo A., Scheibye-Knudsen M., Song S., He H.-J., Armour S.M., Hubbard B.P., Bohr V.A., Wang L. (2011). Negative regulation of STAT3 protein-mediated cellular respiration by SIRT1 protein. J. Biol. Chem..

[34] Li L., Wei W., Zhang Y., Tu G., Zhang Y., Yang J., Xing Y. (2015). SirT1 and STAT3 protect retinal pigmented epithelium cells against oxidative stress. Mol. Med. Rep..

[35] Ciceri P., Artioli L., Magagnoli L., Barassi A., Alvarez J.-C., Massy Z.A., Galassi A., Cozzolino M. (2022). The Role of Uremic Retention Solutes in the MIA Syndrome in Hemodialysis Subjects. Blood Purif..

[36] Rosner M.H., Reis T., Husain-Syed F., Vanholder R., Hutchison C., Stenvinkel P., Blankestijn P.J., Cozzolino M., Juillard L., Kashani K. (2021). Classification of uremic toxins and their role in kidney failure. Clin. J. Am. Soc. Nephrol..

[37] Vanholder R., De Smet R., Glorieux G., Argilés A., Baurmeister U., Brunet P., Clark W., Cohen G., De Deyn P.P., Deppisch R. (2003). Review on uremic toxins: Classification, concentration, and interindividual variability. Kidney Int..

[38] Andrade-Oliveira V., Foresto-Neto O., Watanabe I.K.M., Zatz R., Câmara N.O.S. (2019). Inflammation in Renal Diseases: New and Old Players. Front. Pharmacol..

[39] Zambom F.F.F., Oliveira K.C., Foresto-Neto O., Faustino V.D., Ávila V.F., Albino A.H., Arias S.C.A., Volpini R.A., Malheiros D.M.A.C., Camara N.O.S. (2019). Pathogenic role of innate immunity in a model of chronic NO inhibition associated with salt overload. Am. J. Physiol. Physiol..

